# HPV Infection in Esophageal Squamous Cell Carcinoma and Its Relationship to the Prognosis of Patients in Northern China

**DOI:** 10.1155/2014/804738

**Published:** 2014-01-12

**Authors:** Fangli Cao, Hui Han, Fang Zhang, Baozhong Wang, Wei Ma, Yanwen Wang, Guiming Sun, Miao Shi, Yubo Ren, Yufeng Cheng

**Affiliations:** ^1^Department of Radiation Oncology, Qilu Hospital of Shandong University, No. 44 Wenhua Xi Road, Jinan 250012, China; ^2^Department of Oncology, Liaocheng People's Hospital, Liaocheng 252000, China; ^3^Department of Oncology, The First Hospital of Zibo, Zibo 255000, China; ^4^Department of Radiation Oncology, Cancer Hospital, General Hospital of Ningxia Medical University, Yinchuan 750000, China; ^5^Department of Ultrasound, Liaocheng People's Hospital, Liaocheng 252000, China; ^6^Department of Pathology, Liaocheng People's Hospital, Liaocheng 252000, China

## Abstract

*Purpose*. Human papillomavirus (HPV) as a risk factor for esophageal squamous cell carcinoma (ESCC) has previously been studied, but importance of HPV status in ESCC for prognosis is less clear. *Methods*. A total of 105 specimens with ESCC were tested by in situ hybridization for HPV 16/18 and immunohistochemistry for p16 expression. The 5-year overall survival (OS) and progression-free survival were calculated in relation to these markers and the Cox proportional hazards model was used to determine the hazard ratio (HR) of variables in univariate and multivariate analysis. *Results*. HPV was detected in 27.6% (29) of the 105 patients with ESCC, and all positive cases were HPV-16. Twenty-five (86.2%) of the 29 HPV-positive tumors were stained positive for p16. HPV infected patients had better 5-year rates of OS (65.9% versus 43.4% among patients with HPV-negative tumors; *P* = 0.002 by the log-rank test) and had a 63% reduction in the risk of death (adjusted HR = 0.37, 95% CI = 0.16 to 0.82, and *P* = 0.01). *Conclusions*. HPV infection may be one of many factors contributing to the development of ESCC and tumor HPV status is an independent prognostic factor for survival among patients with ESCC.

## 1. Introduction

Esophageal cancer is the eighth most common cancer and the sixth most common cause of death from cancer, worldwide [1, 2]. Once developed, esophageal cancer usually rapidly invades surrounding tissues and lymph nodes [3]. Due to the absence of early symptoms, invasiveness of the disease, and its late diagnosis, it is generally associated with a poor prognosis [4]. Despite increasing rates of esophageal adenocarcinoma in many western countries, esophageal squamous cell carcinoma (ESCC) remains the dominant histological type of esophageal cancer worldwide. ESCC is still the main cancer burden and the fourth most common cause of death in China [5], especially northern China [6], and thus is the focus of this study.

The etiology of ESCC remains unclear, and epidemiological studies suggest that tobacco smoking, heavy alcohol drinking, micronutrient deficiency [7, 8], and dietary carcinogen exposure may cause the malignancy. Infectious agents have been implicated, as either direct carcinogens or promoters. In particular, human papillomavirus (HPV) has been postulated as a possible cause of ESCC [9]. HPV infection in esophageal cancer was first suggested in 1982 based on histological observations [10]. Subsequent studies using various methods have confirmed the presence of HPV in ESCC [9, 11].

HPV types 16 and 18 are known to cause the majority of squamous cell carcinomas of the cervix [12–15] and are strongly associated with cancers of the head and neck, particularly the oropharynx [16–18]. The viral oncogene products E6 and E7 play a key role in HPV-associated carcinogenesis, abrogating p53 and retinoblastoma tumor suppressor functions, respectively [19, 20]. E7 binds to and degrades Rb, releasing E2F, leading to p16^INK4A^ overexpression, hereafter denoted as p16, which is associated with superior clinical outcome [21, 22]. Thus HPV-positive tumors are characterized by high expression of p16 [23–25] and p16 is widely considered a surrogate marker for HPV infection in the context of squamous cell carcinoma [21, 25].

Some retrospective clinical studies have consistently proved that patients with HPV-positive head and neck squamous cell carcinoma had a better prognosis than patients with HPV-negative tumors [21, 26–28]. Esophagus can be infected with these viruses in the same way as the oral cavity, tonsils, and pharynx; it is supposed that the histological similarities between the head and neck squamous epithelia and esophagus would suggest a similar association and clinical characteristics. The prognostic value of the HPV status has previously been investigated in patients with ESCC. However, the results are much controversial [29–31].

With the present study, we aim to determine the prevalence of HPV infection in ESCC and evaluate its clinical significance. We also sought to evaluate the effect of tumor HPV status on survival of patients with ESCC in northern China.

## 2. Patients and Methods

### 2.1. Patients and Tissues Samples

A total of 279 patients with primary esophageal carcinoma treated with surgery, admitted to the Oncology Center, Qilu Hospital of Shandong University, were identified from December 2006 to January 2008. This hospital is located in Shandong province, which was a high-incidence area for ESCC in China [32]. Patients treated with neoadjuvant therapy, which could potentially interfere with the prevalence of HPV, were excluded, as were patients who died within 30 days after surgery. The additional exclusion criteria comprised the nonsquamous cell subtype and uncooperative patients unable to answer questions or who could not be contacted. A total of 184 patients met the protocol study criteria. All patients provided their written informed consent regarding this study, and the protocol was approved by the Ethics Committee of Qilu Hospital of Shandong University (documentation no. 2012178). Attempts were made to retrieve paraffin blocks from pathology laboratories at which the patients were diagnosed. Of these, pathology review established that samples from 105 patients had sufficient ESCC tumor tissue to detect HPV and p16. Serial 4 *μ*m sections were cut from each patient's tumor tissue. One representative section was stained with hematoxylin and eosin (H&E) to ensure the tissue derived from esophageal cancer. The other sections were prepared for detection. All slides were reviewed by a pathologist specializing in gastrointestinal pathology.

### 2.2. Followup

Postoperative follow-up data were obtained from all patients. The following parameters were studied: gender, age, tumor location, postoperative pathological T and N status (pT and pN), TNM staging according to American Joint Committee on Cancer TNM staging system, differentiation grade of the tumor, adjuvant therapy (postoperative radiotherapy with or without chemotherapy), and smoking and alcohol habits. Anatomical localization of the tumor was grouped into an upper part (15–24 cm), a middle part (25–34 cm), and a lower part of the esophagus (35–46 cm). The tumor status was characterized into localized (primary tumor with or without local node metastases) or advanced disease (with distant metastases). Alcohol intake cutoff point was 0.025 kg/day. The cutoff value was based on the 2011 Chinese Inhabitant Dietary Guideline.

All patients had a regular follow-up schedule including a complete history and physical examination every 3 months during the first 2 years after surgery and every 6 months thereafter. Routine radiological examinations and esophagoscopy were performed when necessary. Patients were followed until death or for a maximum of 5 years.

### 2.3. HPV Detection

All specimens were evaluated for HPV-16 and HPV-18 with using the in situ hybridization-catalyzed signal amplification method for biotinylated probes (GenPoint, Dako). Briefly, sections underwent conventional deparaffinization, heat-induced target retrieval was performed, and digestion using proteinase K and then HPV-16 biotinylated DNA probe (GenPoint, Dako) was applied. Sections were then denatured and stained with diaminobenzidine detection system. Sections were counterstained with hematoxylin. All tumors were further evaluated for HPV-18 by means of HPV-18 biotinylated DNA probe (GenPoint, Dako). The positive control was a cervical squamous cell carcinoma sample, whereas the negative control was obtained by omitting the HPV probe. All slides were scored as positive or negative. Brown staining confined to nuclei of infected tumor cells was defined positive. All scorings were conducted with no knowledge of p16 immunohistochemistry status.

### 2.4. P16^INK4A^ Immunohistochemistry

P16 immunohistochemical detection was done as described previously [33]. Briefly, after formalin-fixed, paraffin-embedded tumor specimens were deparaffinized, antigen retrieval was performed by use of heat-induced epitope retrieval with Tris-EDTA (PH = 9.0, Dako) according to the manufacturer's recommendations. Processions were carried out by the Dako Envision-System method (code: GK500705) using a primary antibody against p16 (monoclonal mouse anti-human p16^INK4A^ protein, Clone G175-405, Dako). A p16-positive tumor was used as a positive control; negative controls were obtained by omitting the primary antibody. P16-positive was defined as >50% of cells showing strong nuclear and cytoplasm immunolabeling. All scorings were conducted with no knowledge of clinical characteristics or outcome.

### 2.5. Statistical Analysis

All analyses were performed using SPSS 16 (SPSS Inc., Chicago, IL). Statistical analyses included univariate analyses of demographic and postoperative outcome data. For these analyses, the differences between the groups were tested for significance using the Mann-Whitney test for continuous variables and the chi-square test or Fisher's exact test for categorical variables and the Kruskal-Wallis test for ranked data. The Kaplan-Meier method and log-rank test were used for analysis and comparison of survival curves. The primary end point was overall survival (OS), defined as the time from date of surgery to death. Secondary end points included progression-free survival (PFS), defined as the time from date of surgery to death or the first documented relapse, which was categorized as local-regional disease (tumor at the primary site or regional nodes) or distant metastases. Death from the primary cancer without a documented site of recurrence or death from an unknown cause was considered death from local-regional disease. PFS and its components were adopted to facilitate comparison with published meta-analyses [34]. The Cox proportional hazards model was used to determine the hazard ratio (HR) of variables on 5-year OS and PFS in univariate and multivariate analysis. The results were given as HRs with their 95% confidence interval (CI). *P* values less than 0.05 were considered statistically significant.

## 3. Results

### 3.1. Patient Characteristics

A total of 105 patients (84 males and 21 females) met the protocol study criteria for analysis. The median age of the patients was 60 (range: 42–78) years at the date of surgery. Patients were divided into two groups according to the tumor HPV status. Baseline characteristics of HPV-positive and HPV-negative patients are shown in Table 1. There were no significant differences between the groups with respect to gender (*P* = 0.66), age (*P* = 0.22), pT status (*P* = 0.18), pN status (*P* = 0.27), TNM stage (AJCC) (*P* = 0.14), differentiation grade (*P* = 0.21), adjuvant therapy (*P* = 0.41), smoking (*P* = 0.13), and alcohol consumption (*P* = 0.78) and only marginally associated with tumor location (*P* = 0.07).

### 3.2. Analysis of HPV and p16

**Figure 1:**
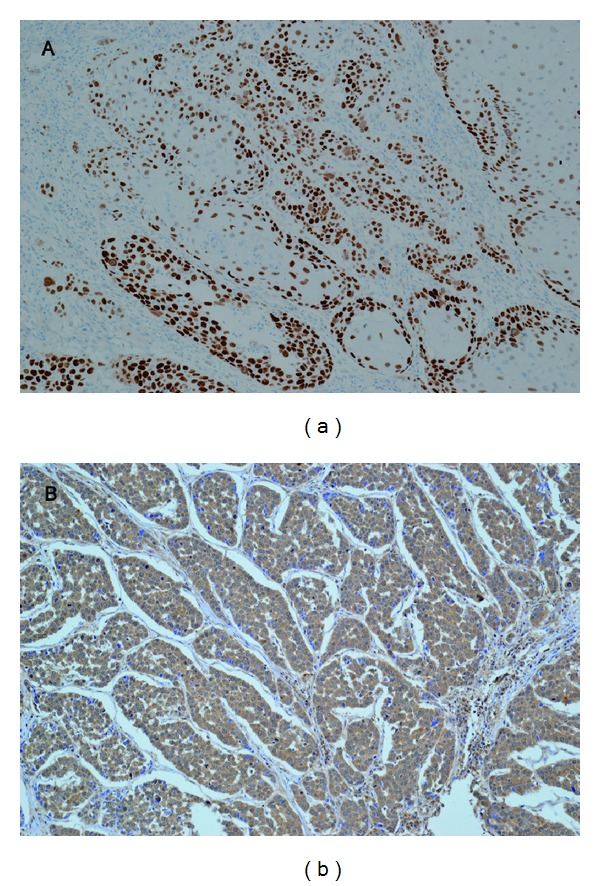
(a) In situ hybridization signal of HPV-positive esophageal squamous cell carcinomas. Numerous tumor cells show positive nuclear signals. (b) Immunohistochemical staining of p16^INK4A^ in esophageal squamous cell carcinomas. More than 50% of tumor cells showing strong nuclear and cytoplasm immunolabeling. (Original magnification ×200.)

Twenty-nine (27.6%) of the 105 ESCC patients were determined to be HPV-positive by in situ hybridization, and all positive cases were HPV-16 (Figure 1(a)); none were positive for HPV-18 DNA. The median age of the HPV-positive group was 60 years (range: 44–75 years) and 62 years (range: 42–78 years) in the HPV-negative group. Twenty-five (86.2%) of 29 HPV-positive tumors were stained positive for p16 with immunohistochemistry (Figure 1(b)). P16 expression was strongly associated with HPV positivity (86.2% in HPV-positive tumors versus 18.4% in HPV-negative tumors, *P* < 0.001) (Table 2).

### 3.3. Survival Analysis

**Figure 2:**
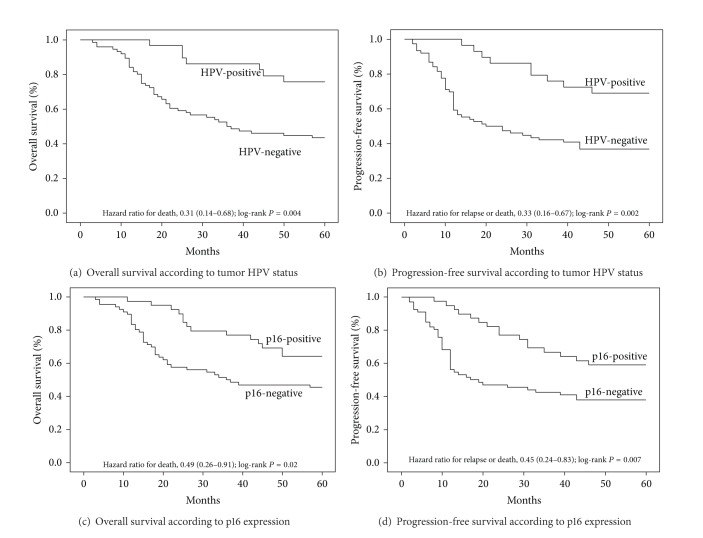
Kaplan-Meier estimates of survival among the study patients, according to tumor HPV status or p16-expression status. For 5-year overall survival rate (a) and 5-year progression-free survival rate (b), HPV was significantly associated with improved outcomes (*P* = 0.002, *P* = 0.001, resp.). For 5-year OS rate (c) and 5-year progression-free survival rate (d), p16 was significantly associated with improved outcomes (*P* = 0.021, *P* = 0.007, resp.).

Based on Kaplan-Meier analysis, patients with HPV-positive tumors had better survival than patients with HPV-negative ones (*P* = 0.002, log-rank test). The 5-year rates of OS were 65.9% in the HPV-positive subgroup and 43.4% in the HPV-negative one (Figure 2(a)). HPV-positive patients also had statistically significantly better PFS than HPV-negative patients (*P* = 0.001, log-rank test). The 5-year rates of PFS were 61.8% and 36.8%, respectively (Figure 2(b)). Tumors were evaluated for the expression of not only HPV but also a known biomarker of HPV oncoprotein function, the cyclin-dependent-kinase inhibitor p16, which is minimally detectable in HPV-negative tumors [35]. The presence of HPV and p16 expression in tumors had a good agreement (kappa = 0.61; 95% CI: 0.45 to 0.77). Using p16 expression as a stratification factor, we found differences in OS and PFS that were consistent with those based on HPV status. The 5-year rates of OS were 64.1% in the subgroup that was positive for p16 expression and 45.5% in the negative subgroup (*P* = 0.021, log-rank test) (Figure 2(c)). The 5-year rates of PFS were 58.7% and 37.9%, respectively (*P* = 0.007, log-rank test) (Figure 2(d)).

Univariate analysis was performed to evaluate factors potentially associated with OS and PFS (Table 3). Gender, age, tumor location, differentiation grade of the tumor, adjuvant therapy, and alcohol habits were not important determinants of survival or PFS. However, pT and pN status, TNM staging, and smoking and tumor HPV status were associated with OS or PFS. T status (T1/T2 versus T3/T4, HR = 3.44, and 95% CI = 1.85 to 6.40), N status (N0 versus N1/N2/N3, HR = 2.71, and 95% CI = 1.55 to 4.73), TNM stage (AJCC stage I/II versus III/IV, HR = 3.04, and 95% CI = 1.74 to 5.32), and tumor HPV status (positive versus negative, HR = 3.26, and 95% CI = 1.46 to 7.25) were associated with OS. T status (T1/T2 versus T3/T4, HR = 2.42, and 95% CI = 1.40 to 4.19), N status (N0 versus N1/N2/N3, HR = 2.79, and 95% CI = 1.66 to 4.72), TNM stage (AJCC stage I/II versus III/IV, HR = 2.66, and 95% CI = 1.57 to 4.50), and tumor HPV status (positive versus negative, HR = 3.01, and 95% CI = 1.50 to 6.17) were associated with PFS. The association of tumor HPV status with survival could not be explained by smoking: patients with HPV-positive tumors with or without a history of smoking had a similar reduction in risk of mortality when compared with their HPV-negative counterparts. Tobacco smoking was also associated with OS and PFS both in the subgroup of patients (<20 versus ≥20, HR = 1.88, and 95% CI = 1.03 to 3.45 and HR = 1.96 and 95% CI = 1.11 to 3.45, resp.).

We then performed multivariable analysis to estimate the association of tumor HPV status with survival outcomes (Table 4). In this analysis, T status (T1/T2 versus T3/T4, adjusted HR = 2.65, 95% CI = 1.39 to 5.05, and *P* = 0.003), N status (N0 versus N1/N2/N3, adjusted HR = 2.07, 95% CI = 1.16 to 3.72, and *P* = 0.01), and TNM stage (I/II versus III/IV, adjusted HR = 1.91, 95% CI = 0.28 to 2.43, and *P* = 0.04) were associated with degraded mortality risk after adjustment for smoking and tumor HPV status. Tumor HPV status was independently associated with mortality risk after adjustment for pT status, pN status, TNM stage, and smoking: patients with HPV-positive tumors had a 63% lower risk of death than patients with HPV-negative (adjusted HR = 0.37, 95% CI = 0.16 to 0.82, and *P* = 0.01). After adjustment for pT status, pN status, TNM stage, and smoking, tumor HPV status was also statistically significantly associated with PFS. Patients with HPV-positive tumors had a risk of progression that was 62% lower than that of patients with HPV-negative tumors (adjusted HR = 0.38, 95% CI = 0.18 to 0.77, and *P* = 0.008).

## 4. Discussion

HPV is a small double-stranded DNA virus with tropism for the squamous epithelium where it can cause hyperproliferative lesions [19]. There are more than 130 HPV types identified and these have been classified into low- or high-risk groups according to their potential for oncogenesis [36]. The high-risk HPV types are closely related to malignancies. According to previous studies, HPV-16 is the most prevalent type in squamous cell carcinoma, followed by HPV-18 [37], while other high-risk HPV types are rare [38, 39].

The etiological role of HPV in ESCC is still unclear. The incidence of HPV in ESCC varies between different geographical areas [9]. It is postulated that areas with high incidence of esophageal carcinoma have higher rates of HPV than areas with low incidence of esophageal carcinoma [40]. In our study, we observed an association between HPV infection and ESCC, HPV was detected in 27.6% of the cases by the use of in situ hybridization, and all cases were HPV-16 positive. The observation was consistent with the previous studies in high-risk areas for ESCC in China [29, 39, 41, 42].

In the present study, we also found that there were marginally significant differences between the HPV-positive and -negative ESCC (*P* = 0.07) with tumor location. HPV infection in upper esophagus was higher than lower. Potentially possible causes were the route of HPV infection and the histological similarities between the oropharyngeal squamous epithelia and upper digestive tract. HPV is currently one of the most common sexually transmitted infections worldwide [43]. Numerous studies have examined that changes in sexual behavior may be able to explain the increase in the incidence of HPV-positive cancers [44, 45]. Esophagus can be infected with these viruses in the same way as the oral cavity, tonsils, and pharynx.

In our study, 25 (86.2%) of the 29 HPV-positive ESCC cases expressed p16, while 14 (18.4%) of 76 HPV-negative subgroup. We observed strong agreement between tumor HPV status by in situ hybridization and p16 by immunohistochemistry, an established biomarker for the function of the HPV E7 oncoprotein. HPV in situ hybridization assay has sensitivity for single viral copies, and a positive result is strongly correlated with expression of the HPV E6 and E7 oncogenes which is the standard for defining a tumor as being effected with HPV [46, 47]. A restriction of our study is not to detect the other subtypes except HPV-16/18, the misclassification of HPV-positive tumors, as HPV-negative tumors probably emerge. The expression of p16 is not specific for HPV type; therefore, p16 immunohistochemistry is a very good surrogate marker of HPV infection for ESCC.

The prognostic value of HPV status has previously been investigated in patients with ESCC. However, the results were much controversial. Furihata et al. reported that HPV-positive patients had worse survival than HPV-negative patients with an overexpression of p53 in esophageal carcinoma patients; they concluded that HPV infection and p53 overexpression indicate poor prognosis [30]. Hippeläinen et al. reported that HPV were involved in 11% of 61 patients with ESCC but without prognostic value [29]. Dreilich et al. reported patients with a HPV-16 viral load > 1.0 viral genome per cell had higher survival rates compared to patients with a HPV-16 viral load < 1.0 viral genome per cell [31].

On the basis of our data, tumor HPV status was an independent prognostic factor for OS and PFS among patients with ESCC. Other retrospective researches have also consistently demonstrated that patients with HPV-positive tumors have a superior prognosis than patients with HPV-negative ones [23, 28, 48, 49]. Several hypotheses have been proposed to explain these results. Cisplatin sensitivity is increased in HPV-16 transfected ovarian cancer cells in vitro studies [50], which imply a better prognosis. HPV-DNA integration is confined to the neoplastic and dysplastic tissue only, so no effect is observed in the field cancerization in HPV-positive tumors [51, 52]. The relationship between the immune system, HPV status, and outcome remains an interesting area of ongoing research; a higher percentage of CD8 cells in the peripheral blood and a lower CD4/CD8 ratio and higher mean sum of CD4 and CD8 infiltrates in the tumor microenvironment may be predictive of better outcome [53]. Integration of HPV results in higher expression of the oncoproteins E6/E7, thereby abrogating the p53 and Rb protein functions, promoting genomic rearrangements [54]; rearranged DNA is theoretically more sensitive to radiation and chemotherapy, providing an explanation for the indication of higher survival rates for patients with HPV-positive tumors. Therefore, the biologic basis for the improved survival among the HPV-positive patients is unclear and warrants further study.

Smoking is associated with an increased risk of ESCC [55] and a poor outcome [22]. In the present study, patients with pack-years of smoking < 20 indicated a trend towards better survival than patients with pack-years of smoking ≥ 20, although this was not statistically significant (*P* = 0.06). However, patients with pack-years of smoking < 20 had a 49% reduction in their risk of progression compared with patients with pack-years of smoking ≥ 20 (*P* = 0.02). The link between HPV positivity in ESCC and smoking was still under investigation. Some studies have suggested synergistic effect [16, 56] while others have not [18, 57]. Genetic alterations induced by tobacco-associated carcinogens may be strengthened by HPV and cause HPV-positive tumors less sensitive to treatment. Our sample was too small to exclude confounding by smoking, although there was no apparent difference in the prevalence of smoking intake among patients with HPV-positive versus HPV-negative. Analysis of a larger study could more thoroughly evaluate the possibility of confounding by smoking via analysis of different levels of tobacco consumption.

Additional variables of potential prognostic importance, such as weight loss, anemia, performance status, dietary habits, and sexual behavior, were lacking in our study. Sample size limits the number of variables that could be included in our models. Factors not included in our models may be important and affect survival. Although statistically significant differences in survival were observed between HPV-positive and -negative, definitive conclusions cannot be drawn from this study due to its small sample size and retrospective nature; larger confirmatory studies are needed to provide definitive evidence.

In addition, the temporal sequence of HPV infection and onset of ESCC cannot be ascertained. Therefore, a causal relationship between exposure and outcome must be a tentative one, despite the association of infection and tumor which has been observed. A prospective study would be needed to further address this issue. The role of viruses has great potential in the clinical practice, particularly when investigated in combination with other factors. This study provides a direction for future clinical research. However, given the limited sample size, the results of this study should be interpreted with caution.

## Figures and Tables

**Table 1:** Baseline characteristics of the study patients and their tumors, according to tumor HPV status.

Characteristics	Total (*N* = 105) no. (%)	HPV-positive (*N* = 29) no. (%)	HPV-negative (*N* = 76) no. (%)	*P* value
Gender				
Male	84 (80.0)	24 (82.8)	60 (78.9)	0.66
Female	21 (20.0)	5 (17.2)	16 (21.1)	
Age				
Median (range)	60 (42–78)	60 (44–75)	62 (42–78)	0.22^△^
Tumor location				
Cervical/upper	11 (10.5)	6 (20.7)	5 (6.6)	0.07
Middle	49 (46.7)	14 (48.3)	35 (46.1)	
Low	45 (42.9)	9 (31.0)	36 (47.4)	
pT status				
pT1	20 (19.0)	7 (24.1)	13 (17.1)	0.18^*☆*^
pT2	22 (21.0)	7 (24.1)	15 (19.7)	
pT3	58 (55.2)	15 (51.8)	43 (56.6)	
pT4	5 (4.8)	0 (0.0)	5 (6.6)	
pN status				
pN0	68 (64.8)	21 (72.4)	47 (61.8)	0.27^*☆*^
pN1	25 (23.8)	6 (20.7)	19 (25.0)	
pN2	10 (9.5)	2 (6.9)	8 (10.5)	
pN3	2 (1.9)	0 (0.0)	2 (2.6)	
TNM stage (AJCC)				
I	23 (21.9)	7 (24.1)	16 (21.1)	0.14
II	49 (46.7)	17 (58.6)	32 (42.1)	
III	33 (31.4)	5 (17.2)	28 (36.8)	
Differentiation grade				
Well	27 (25.7)	11 (37.9)	16 (21.1)	0.21
Moderate	44 (41.9)	10 (34.5)	34 (44.7)	
Poor	34 (32.4)	8 (27.6)	26 (34.2)	
Adjuvant therapy				
No	61 (58.1)	15 (51.7)	46 (60.5)	0.41
yes	44 (41.9)	14 (48.3)	30 (39.5)	
Pack-years of smoking^*※*^				
<20	42 (40.0)	15 (51.7)	27 (35.5)	0.13
≥20	63 (60.0)	14 (48.3)	49 (64.5)	
Alcohol intake (kg/day)				
<20	53 (50.5)	14 (48.3)	39 (51.3)	0.78
≥20	52 (49.5)	15 (51.7)	37 (48.7)	

AJCC: American Joint Commission on Cancer Staging; Pt: pathological tumor stage; pN: pathological node stage.

^*☆*^ *P* values were calculated with the use of the Kruskal-Wallis test.

^△^ *P* values were calculated with the use of the Mann-Whitney test.

^*※*^A pack-year is defined as the equivalent of smoking one pack of cigarettes per day for 1 year.

**Table 2:** Correlation between HPV in situ hybridization and p16 immunohistochemistry in esophageal squamous cell carcinoma.

p16 status	Total (*N* = 105) no. (%)	HPV-positive (*N* = 29) no. (%)	HPV-negative (*N* = 76) no. (%)	*P* value	Kappa value
Positive	39 (37.1)	25 (86.2)	14 (18.4)	<0.001	0.61
Negative	66 (62.9)	4 (13.8)	62 (81.6)		

*P* and Kappa values were calculated with the use of Pearson's chi-square test and Cohen Kappa test, respectively.

**Table 3:** Cox univariate analysis for 5-year survival and progression-free survival in the study patients with esophageal squamous cell carcinoma.

Parameters	Univariate analysis					
	5-yr overall survival			5-yr progression-free survival		
	HR	95% CI	*P* value	HR	95% CI	*P* value
Gender	1.29	0.68–2.47	0.44	1.32	0.71–2.45	0.38
Male versus female						
Age	0.98	0.56–1.70	0.93	0.85	0.51–1.44	0.55
<60 versus ≥60						
Location	0.75	0.30–1.89	0.54	0.66	0.28–1.55	0.34
Cervical/upper versus middle/low						
Differentiation	1.01	0.56–1.81	0.99	1.12	0.58–2.14	0.74
Well/moderate versus poor						
pT status	3.44	1.85–6.40	<0.001	2.42	1.40–4.19	0.002
T1/T2 versus T3/T4						
pN status	2.71	1.55–4.73	<0.001	2.79	1.66–4.72	0.001
N0 versus N1/N2/N3						
TNM stage	3.04	1.74–5.32	<0.001	2.66	1.57–4.50	<0.001
I/II versus III/IV						
Adjuvant therapy	0.75	0.54–1.53	0.35	0.51	0.45–1.28	0.12
No versus yes						
Pack-years of smoking	1.88	1.03–3.45	0.04	1.96	1.11–3.45	0.02
<20 versus ≥20						
Alcohol intake (kg/day)	1.66	0.95–2.92	0.08	1.61	0.95–2.73	0.06
<0.025 versus ≥0.025						
Tumor HPV status	0.31	0.14–0.68	0.004	0.33	0.16–0.67	0.002
Negative versus positive						
Tumor HPV status	3.26	1.46–7.25	0.004	3.01	1.50–6.17	0.002
Positive versus negative						

HR: hazard ratio; CI: confidence interval; a pack-year: the equivalent of smoking one pack of cigarettes per day for 1 year.

**Table 4:** Multivariate Cox analysis for 5-year survival and progression-free survival in the study patients with esophageal squamous cell carcinoma.

Parameters	Multivariate analysis					
	5-yr overall survival			5-yr progression-free survival		
	HR	95% CI	*P* value	HR	95% CI	*P* value
pT status	2.65	1.39–5.05	0.003	2.09	1.17–3.72	0.01
T1/T2 versus T3/T4						
pN status	2.07	1.16–3.72	0.01	2.14	1.24–3.68	0.006
N0 versus N1/N2/N3						
TNM stage	1.91	0.28–2.43	0.04	0.48	0.15–2.47	0.03
I/II versus III/IV						
Pack-years of smoking	1.84	1.00–3.39	0.06	1.94	1.09–3.44	0.02
<20 versus ≥20						
Tumor HPV status	0.37	0.16–0.82	0.01	0.38	0.18–0.77	0.008
Positive versus negative						

HR: hazard ratio; CI: confidence interval; a pack-year: the equivalent of smoking one pack of cigarettes per day for 1 year.
